# WO_x_ channel engineering of Cu-ion-driven synaptic transistor array for low-power neuromorphic computing

**DOI:** 10.1038/s41598-023-49251-6

**Published:** 2023-12-13

**Authors:** Seonuk Jeon, Heebum Kang, Hyunjeong Kwak, Kyungmi Noh, Seungkun Kim, Nayeon Kim, Hyun Wook Kim, Eunryeong Hong, Seyoung Kim, Jiyong Woo

**Affiliations:** 1 School of Electronic and Electrical Engineering, Kyungpook National University, Daegu, 41566 South Korea; 2 Department of Materials Science and Engineering, Pohang University of Science and Technology, Pohang, 37673 South Korea

**Keywords:** Electrical and electronic engineering, Other nanotechnology

## Abstract

The multilevel current states of synaptic devices in artificial neural networks enable next-generation computing to perform cognitive functions in an energy-efficient manner. Moreover, considering large-scale synaptic arrays, multiple states programmed in a low-current regime may be required to achieve low energy consumption, as demonstrated by simple numerical calculations. Thus, we propose a three-terminal Cu-ion-actuated CuO_x_/HfO_x_/WO_3_ synaptic transistor array that exhibits analogously modulated channel current states in the range of tens of nanoamperes, enabled by WO_3_ channel engineering. The introduction of an amorphous stoichiometric WO_3_ channel formed by reactive sputtering with O gas significantly lowered the channel current but left it almost unchanged with respect to consecutive gate voltage pulses. An additional annealing process at 450 °C crystallized the WO_3_, allowing analog switching in the range of tens of nanoamperes. The incorporation of N gas during annealing induced a highly conductive channel, making the channel current modulation negligible as a function of the gate pulse. Using this optimized gate stack, Poole–Frenkel conduction was identified as a major transport characteristic in a temperature-dependent study. In addition, we found that the channel current modulation is a function of the gate current response, which is related to the degree of progressive movement of the Cu ions. Finally, the synaptic characteristics were updated using fully parallel programming and demonstrated in a 7 × 7 array. Using the CuO_x_/HfO_x_/WO_3_ synaptic transistors as weight elements in multilayer neural networks, we achieved a 90% recognition accuracy on the Fashion-MNIST dataset.

## Introduction

In this era of big data, the amount of data to be processed has increased significantly. Data processing through the conventional von Neumann computing architecture, where the processor and memory units are physically separated, has caused significant latency bottlenecks and power consumption^1^. To address this challenge, brain-inspired neuromorphic systems using parallel-connected biological synapses have been proposed as new computing architectures to enhance computation efficiency^2–5^. To implement this architecture in hardware systems, it is important to emulate the functions of biological synapses using analog memory devices. Among the various candidates for synaptic devices, resistive switching random-access memory (RRAM) has mainly been explored because of its scalability^6–8^. The multiple resistance states of RRAM can be demonstrated by the precise control of the formation and rupture of local conductive filaments through materials engineering and novel programming schemes^9^. However, stochastic ion motion in the switching layer interferes with the filament, causing a nonuniform synaptic response with respect to the programming pulses^10–14^.

**Figure 1:**
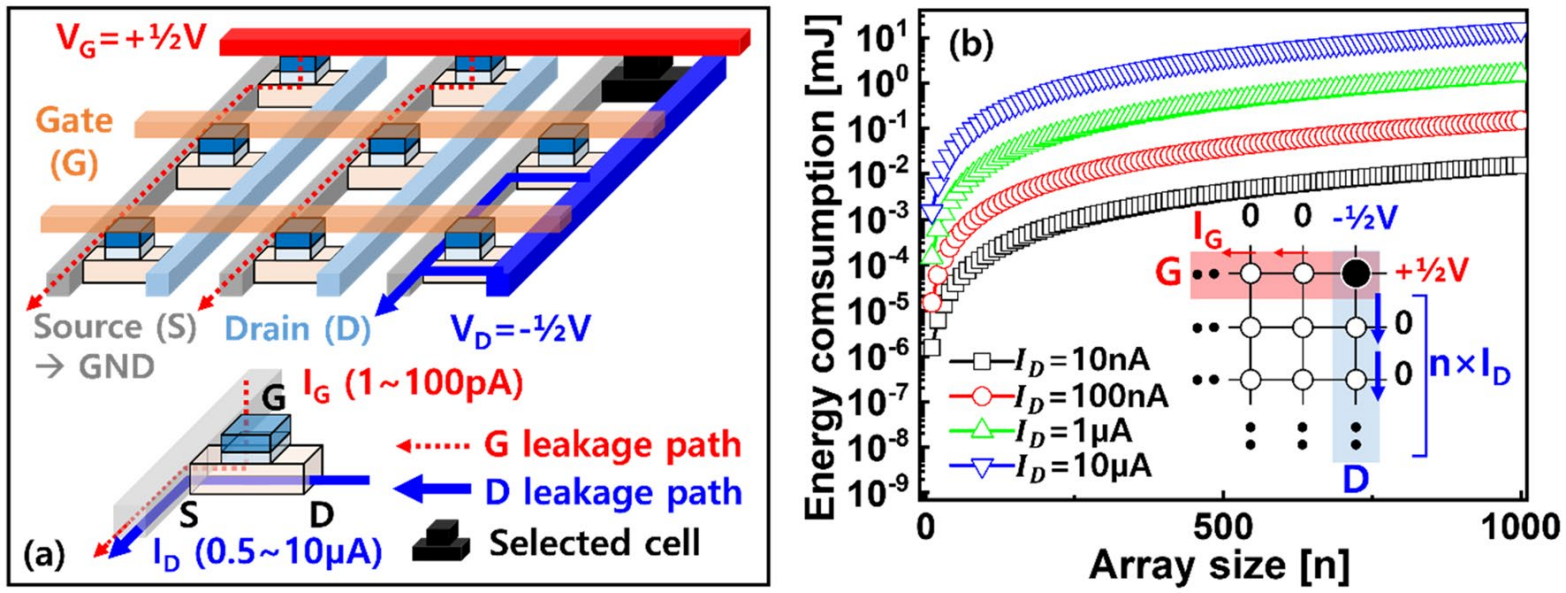
(**a**) Schematic illustration of leakage current sources in synaptic arrays. The half-bias scheme was used to program the farthest cell for simplicity. (**b**) Numerical calculations show the impact of leakage currents on energy consumption with respect to array size. Energy was calculated considering the leakage currents originated from unselected synaptic transistors, where either V_G_ or V_D_ was applied.

For uniform analog synaptic devices, ion-actuated transistors, which have also been referred to as electrochemical random-access memory or redox transistors and were inspired by the field-driven Li ion motion of rechargeable batteries, have recently been suggested^15^. Instead of the gate dielectric of a conventional transistor, an electrolyte that not only contains mobile ion sources but also allows ionic motion driven by the field can be utilized. As the number of ions attracted (or repulsed) to the channel layer increases (or decreases), the channel current (I_D_) between the source (S) and drain (D) is gradually tuned towards higher (or lower) levels as a function of the positive (or negative) programing pulses applied to the gate (G). Thus far, this analog switching behavior has been demonstrated in synaptic transistor unit cells for various combinations of electrolyte/channel material stacks and mobile ion species such as Li, H, O, and Cu ions^15–18^. Recent advances in synaptic transistors have been extended to array-level analyses^19–21^. Analog switching of some or all cells in arrays that can be updated simultaneously has been experimentally achieved using a half-bias scheme, where the drain voltage (V_D_) and gate voltage (V_G_) are used during programming. It is worth noting that the I_D_ of the synaptic transistor should be as low as possible considering the energy consumption at the array level^21^. This is because, unlike usual transistors serving as switch elements, analog synaptic transistors are always on-channel without a threshold voltage required to allow current to flow in the channel. The magnitude of the I_D_ of the synaptic transistor is tuned only by the V_G_. Assuming that only the farthest synaptic transistor in the array is programmed, unselected synaptic transistors located on a horizontal (or vertical) line applied to V_G_ (or V_D_) generate unwanted gate (or drain) leakage currents, as shown in Fig. 1a. In particular, although extremely low gate currents (I_G_s) of several tens of picoamperes have been reported to date, the I_D_ has mainly been several microamperes^15–18,22^. This implies that the magnitude of the I_D_ is primarily related to the energy consumption in the synaptic array and its impact becomes significant as the array size increases, as shown in Fig. 1b.

Therefore, we fabricated a CuO_x_/HfO_x_/WO_x_ synaptic transistor array that exhibited analog switching in the range of tens of microamperes. Next, the effect of the WO_x_ channel layer was investigated using the sputtering method and annealing conditions related to the stoichiometry and crystallinity of the material.

**Figure 2:**
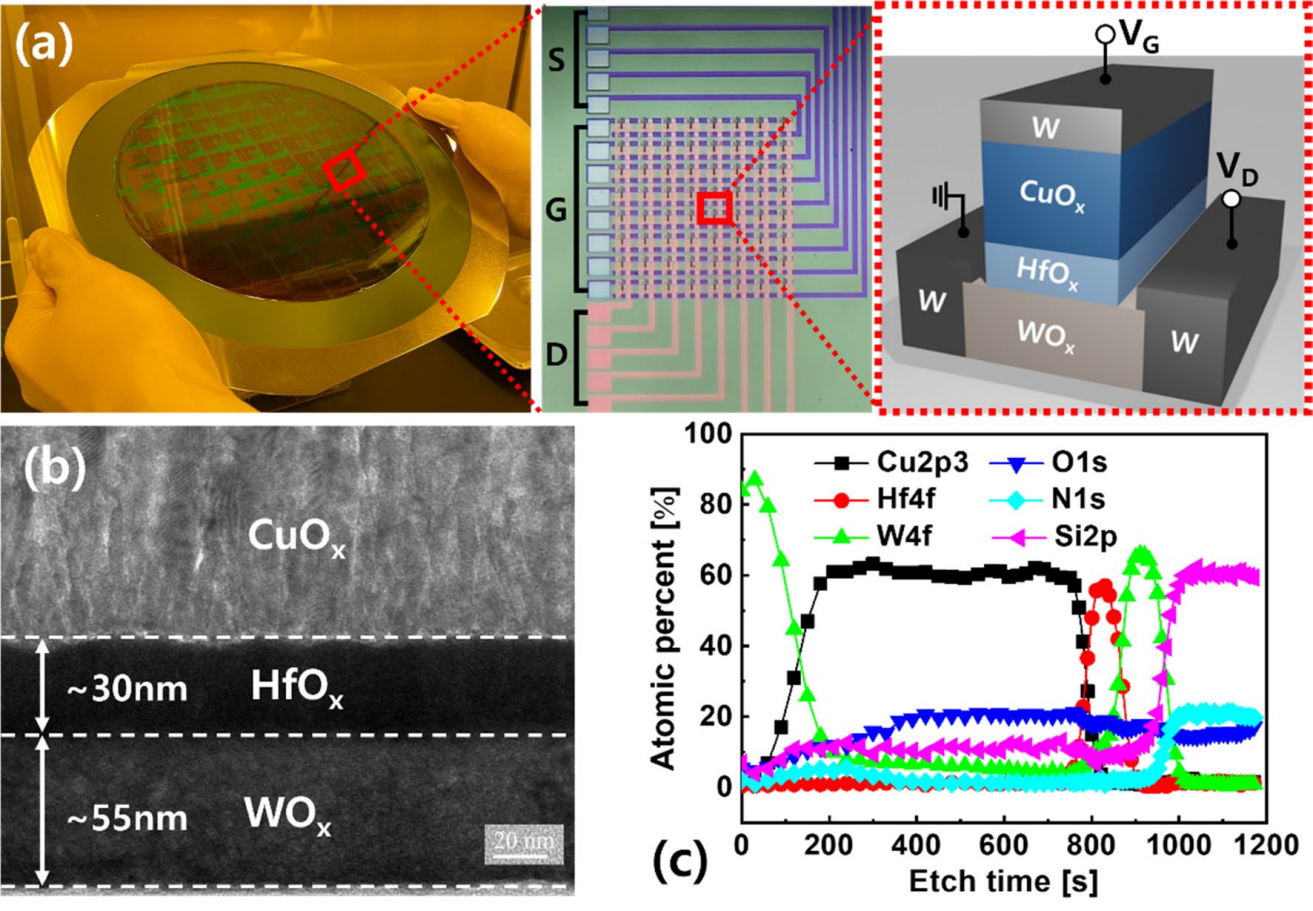
(**a**) Fabricated CuO_x_/HfO_x_/WO_x_ synaptic transistor arrays. I_D_ of the synaptic transistor was updated by applying V_G_ and grounding S. Updated I_D_ was read by addressing voltage to D. (**b**) Cross-sectional TEM image and (**c**) XPS depth profiling of the synaptic transistor.

The Cu-ion-actuated synaptic transistor structure developed in our previous study was used in this study^18^. For the synaptic transistor array, S and D lines consisting of W were patterned on an Si/SiO_2_ wafer, as shown in Fig. 2a. Via-holes for the S/D contacts were defined after Si_3_N_4_ interlayer deposition. A WO_x_ channel with a length (or width) of 20 (or 5) μm was deposited by sputtering with a WO_3_ single target. For comparison, a different channel layer was formed by reactive sputtering with a W metal target under ambient Ar and O plasma conditions. Additional annealing was performed at 450 °C using a rapid thermal annealing system to lower I_D_ in the range of nanoamperes. Subsequently, an HfO_x_ electrolyte and CuO_x_ G with a length of 10 μm were deposited by sputtering using HfO_x_ and Cu targets, respectively. Finally, a W capping layer was formed on top of the G and S/D contacts by sputtering. The thickness of each layer was evaluated by alpha step at the 3-D convergence center of Inha University. The fabricated CuO_x_/HfO_x_/WO_x_ synaptic transistor was analyzed using transmission electron microscopy (TEM) and X-ray photoelectron spectroscopy (XPS) depth profiling, and each layer was clearly distinguished, as shown in Fig. 2b and c.

**Figure 3:**
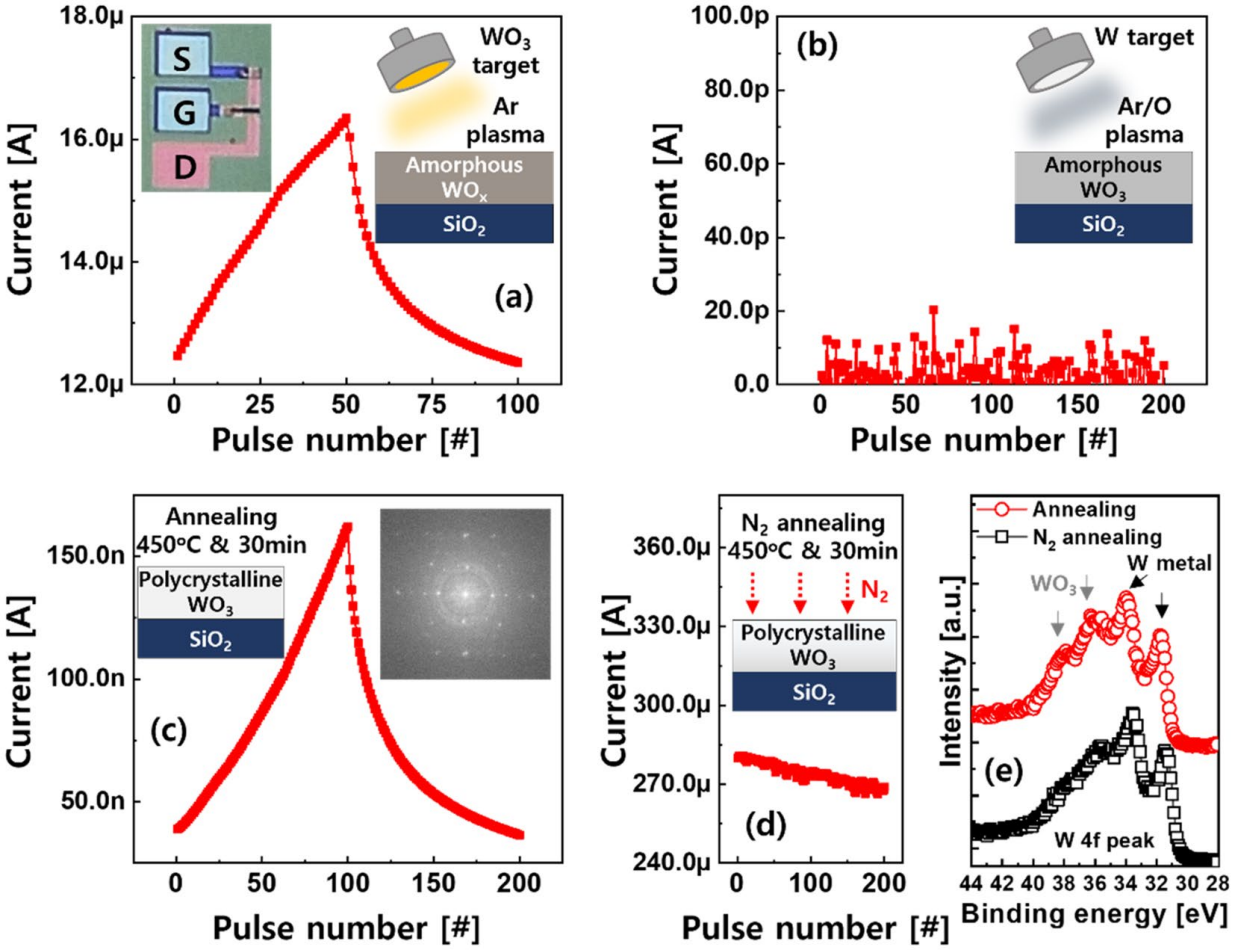
V_G_ of + 6 V (or − 5 V) with a pulse width of 100 ms was used for all devices to evaluate device characteristics. (**a**) Analog synaptic behavior observed in the synaptic transistor employing amorphous WO_x_ channel. (**b**) No switching was observed when relatively stoichiometric WO_3_ channel was used. (**c**) Crystallized WO_3_ channel, which was verified by fast Fourier transformation image, allowed the I_D_ to be adjusted by pulse number. (**d**) Increased I_D_ level due to N annealing hid the switching behavior. (**e**) W 4f peak obtained through XPS analysis.

The electrical characteristics of the CuO_x_/HfO_x_/WO_x_ unit synaptic transistor were evaluated by applying a V_G_ of + 6 V (or − 5 V) with a pulse width of 100 ms to increase (or decrease) the I_D_, as shown in Fig. 3a. The tuned I_D_ was sensed by applying a V_D_ of 0.5 V. The observed switching behavior was attributed to the reversible driving of Cu ions back and forth through the HfO_x_ layer. The effective thickness of HfO_x_ was thinned or thickened, and the electric field-adjusted channel conductivity was enhanced or weakened^23^. As reported in a previous study, a limited number of Cu ions was provided by the CuO_x_ G electrode, which could be effectively and precisely controlled by successive V_G_ pulses to allow linear and symmetric synaptic behavior in the range of tens of microamperes^18^. Under the given programming conditions, we first analyzed the effect of the stoichiometry of the channel layer. A stoichiometric WO_3_ layer was formed by reactive sputtering with O gas injection, as shown in Fig. 3b. Although an extremely low I_D_ at a level below tens of picoamperes was measured, no change in I_D_ was observed as a function of V_G_ pulses. When annealing was conducted at 450 °C and atmospheric pressure for 30 min, I_D_ became higher and began to be analogously controlled by the V_G_ pulses, as shown in Fig. 3c. The WO_3_ layer crystallizes at temperatures above 400 °C^24^. The polycrystalline WO_3_ layer, which was verified by the fast Fourier transform in the TEM analysis (inset of Fig. 3c), boosted the conductivity. Thus, most of the applied V_G_ was transferred to the HfO_x_ electrolyte to drive the Cu ions, leading to a progressive increase and decrease in I_D_ for 200 consecutive V_G_ pulses. However, when ambient N gas was applied during annealing, I_D_ was increased to hundreds of microamperes, making the I_D_ modulation negligible, as shown in Fig. 3d. To identify the impact of the annealing gas on analog switching, the W 4f peak at the WO_3_ channel interface of the two annealed devices, obtained through XPS analysis, was investigated, as shown in Fig. 3e. Two peaks at binding energies of approximately 31 and 34 eV, indicating metallic states related to nonstoichiometric WO_x_ were clearly observed for both samples. However, two peaks corresponding to WO_3_ observed at 36.5 and 38 eV were noticeable for the annealed sample without N gas. This implies that N preferentially bonds to W. W–N bonding is usually represented by metallic peaks (approximately 31 and 34 eV); therefore, the enhanced channel conductivity can be attributed to the conductive WN formed.

**Figure 4:**
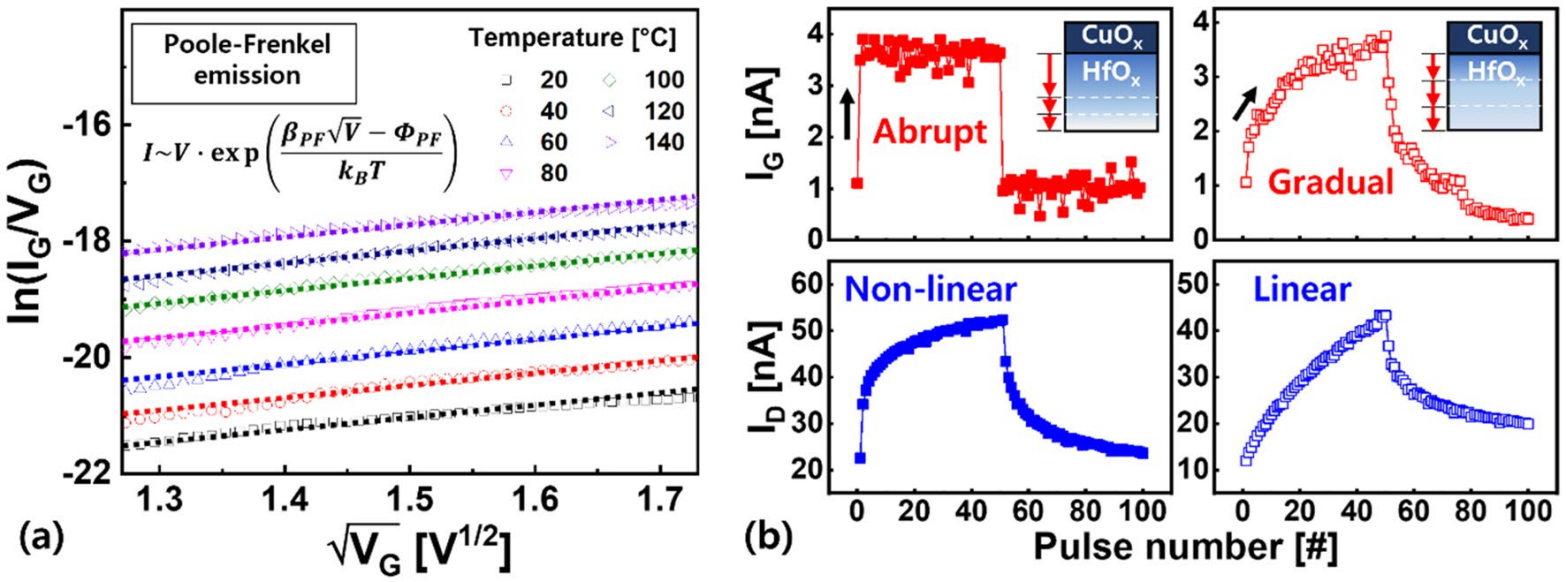
(**a**) Poole–Frenkel conduction of the gate stack. (**b**) Link between the I_G_ response, which can be described by Cu ion motion over the electrolyte, and I_D_ modulation.

Next, we studied the temperature dependence of the I_G_ to identify the mechanisms of analog switching in the optimized gate stack. I_G_, which was obtained by applying voltage to G and grounding S, was measured by raising the temperature to 140 °C. As a result, the transport characteristics were suitably fitted using the Poole–Frenkel emission, as shown in Fig. 4a. This indicates that bulk switching across the entire electrolyte area was involved, rather than metallic conduction, owing to the locally clustered Cu ions. Specifically, by comparing simultaneously obtained I_G_ and I_D_, we found that the I_G_ response to V_G_ pulses was related to I_D_ modulation, as shown in Fig. 4b. That is, the gradual transition of I_G_ by a single V_G_ pulse during initial programming was typically projected onto the lateral I_D_, resulting in a linear I_D_ response in working cell. However, at the given programming condition, nonlinear I_D_ behavior was often observed in non-optimized stacks. When we examined the I_G_ response of these failed cells, the abrupt increase and saturation of I_G_ was observed. These results imply that the extent to which Cu ions move steadily through the electrolyte per V_G_ pulse is related to the linearity and symmetry of I_D_.

**Figure 5:**
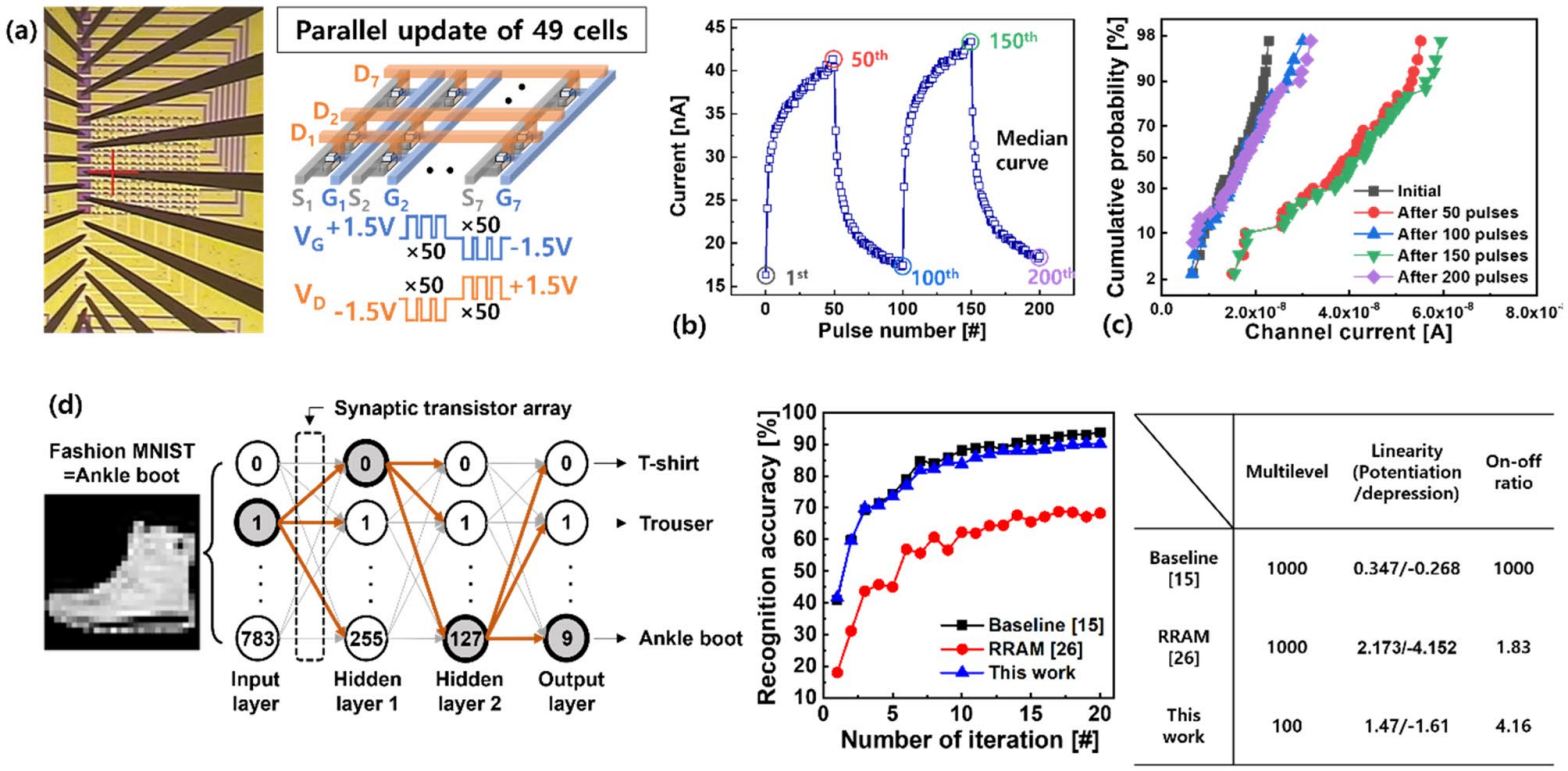
(**a**) Optical microscope image of fabricated synaptic transistor array contacted by probe card. To update all synaptic transistor cells simultaneously, 50 V_G_ (or V_D_) pulses of + 1.5 V (or − 1.5 V) were consecutively addressed to all G (or D) lines to increase I_D_. By changing the polarity of each pulse, I_D_ can be steadily lowered. (**b**) Reliable synaptic behaviors expressed as median were obtained from all cells. (**c**) Distributions of I_D_s when pulses were applied every 50 cycles from the initial state. (**d**) Pattern recognition accuracy of multilayer perceptron evaluated using IBM analog hardware acceleration kit simulator. The parameters used in this simulation were listed in the table.

Finally, we evaluated synaptic arrays employing the engineered WO_3_ channel. As the channel resistance was reduced, a larger electric field across the HfO_x_ layer was used to drive the Cu ions; therefore, analog switching could be achieved by lowering V_G_ to + 3 V. To facilitate fully parallel programming, all the G, S, and D pads of the 7 × 7 array were contacted by a probe card, as shown in Fig. 5a. In the half-bias scheme, the + 3 V (or − 3 V) required to program the unit cell was halved, and + 1.5 V (or − 1.5 V) was simultaneously applied to V_G_ and V_D_. Consequently, a full voltage of approximately ± 3 V was applied to the selected cells. Reliable analog switching behavior was obtained for all synaptic transistors. The cell-to-cell update curves expressed as the median repeatedly increase and decrease, as the polarity of the pulse changes every 50 times for a total of 200 pulses, as shown in Fig. 5b. Specifically, the I_D_ states updated by the first 50 potentiation pulses reached its maximum value and returned to the initial state by the next 50 depression pulses. As a result, the I_D_ states applied at the first, 100th, and 200th pulses (or 50th and 150th) showed uniform cycle-to-cycle distribution, as shown in Fig. 5c. Note that although the reversible analog I_D_ modulation from all cells was achieved, cell-to-cell uniformity in the array needed to be improved. We recently revealed that the effect of Cu ion distribution (or concentration) in the stack on the synaptic behavior through physics-based simulation^25^. Based on the clue, further study is currently underway on process development and strategies to ensure reliability as well as uniformity by controlling the adequate amount of Cu ions in the device.

Based on these results, we built multilayer neural networks with size 784-256-128-10 (from input to output neurons) designed for image recognition, as shown in Fig. 5d. The input signals were transferred from input neurons to output neurons through synaptic weights, which served as the optimized synaptic transistor in this study. The degree of linearity of synaptic characteristics is expressed by a linearity factor, α, which is calculated by the following equation:^26^

$${\text{G}} = { }\left\{ {\begin{array}{*{20}l} {\left( {\left( {G_{MAX}^{{\upalpha }} - G_{MIN}^{{\upalpha }} } \right) \times \omega + G_{MIN}^{{\upalpha }} } \right)^{{\frac{1}{{\upalpha }}}} } \hfill & { \left( {if \alpha \ne 0} \right)} \hfill \\ {G_{MIN} \times \left( {G_{MAX} /G_{MIN} } \right)^{\omega } } \hfill & {\left( {if \alpha = 0} \right)} \hfill \\ \end{array} } \right.,$$

where, G_MAX_ and G_MIN_ are conductance at the maximum and minimum I_D_ state, respectively, and $\omega$ is an internal variable which ranges from 0 to 1. Moreover, α is equal to 1 in the case of the ideal synaptic behavior. Based on these equations, linearity of potentiation (or depression) of 1.47 (or − 1.61) was achieved for the developed synaptic transistor. The difference between the maximum and minimum I_D_ states of ~ 4 was also used in the simulations. Subsequently, the recognition accuracy on the Fashion-MNIST dataset was evaluated with a learning rate of 0.01 using the IBM analog hardware acceleration kit simulator by assuming that the achieved synaptic transistors served as weight elements^26^. Consequently, our device exhibited a recognition accuracy of approximately 90%, which is comparable to the baseline, when the multilevel states were almost linearly adjusted^15,27^.

**Figure 6:**
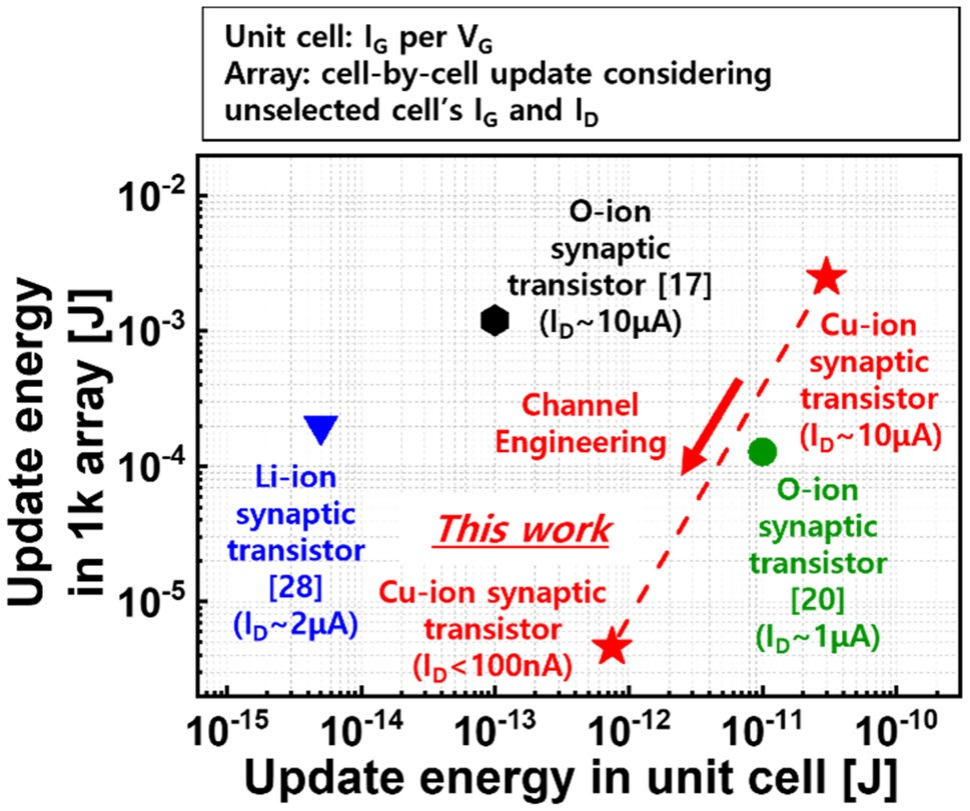
From a unit cell perspective, synaptic transistors that exhibit low I_G_ and are driven by low V_G_ seem to be preferred for low-power application. However, considering update operation in the array, the I_D_ range of the synaptic transistors contributes more to energy consumption.

Finally, through simple numerical analysis, we showed that update energy consumption is reduced by lowering I_D_ of the synaptic transistors, which are assumed to be configured in 1 K array, as shown in Fig. 6. Compared to reported synaptic transistors that utilize the Li or O ions, the V_G_ to drive the Cu ions seemed to be larger. Current has a greater impact on power than voltage because its magnitude can be changed significantly. To date, the energy consumed to change the I_D_ state by a single V_G_ pulse has been mainly discussed considering unit cell operation^28^. However, to sequentially update all cells in the array, the V_G_ and V_D_ signals were applied to the array via all interconnect lines. Thus, synaptic transistors with tunable I_D_ in the low current regime enable low-power operation because the I_D_ is typically higher than the I_G_. In addition to energy consumption, recent study showed that recognition accuracy is degraded due to interference from high I_D_s of neighboring cells^29^. These results mean that the range of I_D_, which has often been ignored, should be considered when designing a three-terminal synaptic transistor.

In this work, we achieved analog switching in a low I_D_ regime (< 100 nA) of a 7 × 7 CuO_x_/HfO_x_/WO_3_ synaptic transistor array using WO_3_ channel engineering. Specifically, we revealed that the I_D_ range could be lowered or increased by utilizing a stoichiometric WO_3_ layer or by strengthening the crystallinity. In addition, the steady and gradual motion of the Cu ions across the gate stack enabled a linearly tuned I_D_ response. The obtained synaptic behavior not only accurately inferred the Fashion-MNIST dataset but also performed recognition with low energy consumption.

## Data Availability

The data that support the findings of this study are available from the corresponding author upon reasonable request.
